# Herpetic Keratouveitis: Missed Diagnosis Leading to Corneal Perforation

**DOI:** 10.7759/cureus.55471

**Published:** 2024-03-04

**Authors:** Kirupakaran Arun, Panagiotis Georgoudis

**Affiliations:** 1 Ophthalmology, Whipps Cross Hospital, London, GBR

**Keywords:** acyclovir, penetrating keratoplasty, iris atrophy, corneal perforation, herpetic anterior uveitis

## Abstract

Herpetic uveitis is an easy diagnosis to miss, which can lead to devastating consequences. The aim of this report is to create awareness of how this disease can present, appropriate clues to the diagnosis, and how it should be managed. We report a case of a 70-year-old female who presented with redness and painless blurry vision in her right eye and was treated with topical corticosteroid drops for presumed idiopathic anterior uveitis. Despite initial symptomatic improvement, she reattended with a significant deterioration in vision and was found to have a large corneal infiltrate and associated perforation. The perforation was sealed with corneal gluing, and she was treated for presumed herpetic anterior uveitis with oral acyclovir. Corneal polymerase chain reaction (PCR) specimen was positive for herpes simplex virus DNA. The perforation started to leak again despite repeat corneal gluing, so an emergency therapeutic penetrating keratoplasty was performed. She has remained on prophylactic oral acyclovir for the last 24 months, with no recurrence and the graft remains clear.

## Introduction

Anterior uveitis is a common presentation to the eye emergency department. Eighty percent of anterior uveitis cases are idiopathic or associated with HLA-B27 positivity [1] and are effectively treated with tapering topical corticosteroids.

Herpes simplex virus (HSV) anterior uveitis is the most common form of infectious anterior uveitis and accounts for 5% of all uveitis cases [2]. It can be challenging to differentiate HSV anterior uveitis from other non-infectious causes of anterior uveitis, especially when there is an absence of other ocular signs of herpetic disease, such as keratitis or periocular dermatitis.

Patients with HSV anterior uveitis frequently present with unilateral redness and photophobia [3]. Examination findings include reduced corneal sensation, anterior chamber inflammation, keratic precipitates, acutely raised intraocular pressure, and patchy iris atrophy [4]. Despite investigations, such as polymerase chain reaction (PCR) of corneal and/or aqueous fluid samples used to confirm the diagnosis [5], it is clear that HSV anterior uveitis remains a clinical diagnosis.

In this report, we present a case of HSV anterior uveitis that was missed at the initial presentation and led to the unfortunate complication of corneal perforation. This was subsequently managed appropriately, an emergency penetrating keratoplasty was performed, and appropriate prophylactic treatment meant that there have been no further recurrences of herpetic ocular disease and the graft has remained clear. This case emphasizes the importance of a clear understanding of diagnostic features that are suggestive of HSV anterior uveitis to initiate appropriate and prompt treatment.

## Case presentation

A 70-year-old female presented with a four-day history of right eye redness and painless reduced vision. There was no history of previous contact lens use. On examination, the best-corrected visual acuity was 20/80 in the right eye and 20/30 in the left eye. A slit lamp examination of the left eye was unremarkable. The right eye was found to have raised intraocular pressure (32 mmHg), mild conjunctival injection, 2+ anterior chamber cells, and inferior keratic precipitates. There was a quiet vitreous and healthy optic disc, macula, and peripheral retina. 

Based on the history and clinical examination, an initial diagnosis of idiopathic acute anterior uveitis was made, and she was started on tapering dexamethasone 0.1% drops over six weeks.

**Figure 1:**
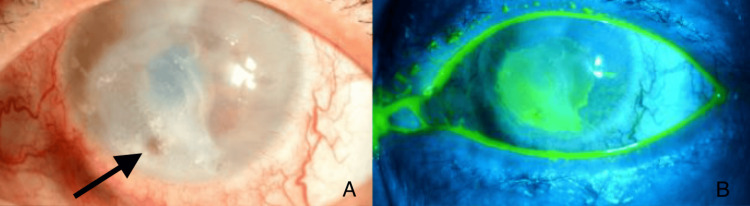
A) Iris tamponade of a corneal perforation within a larger corneal infiltrate. B) Fluorescein staining of the same eye, confirming a large overlying epithelial defect. Slit lamp examination photos of a different patient with the same presentation [4].

However, four weeks later, she re-presented to our eye emergency department with a sudden drop in vision in the right eye and significant watering but minimal pain. On examination, visual acuity had deteriorated to perception of light in the right eye and 20/30 in the left eye. Intraocular pressures were 3 mmHg in the right eye and 14 mmHg in the left eye. The slit lamp examination of the left eye remained unremarkable. However, the right eye showed a significant injection of the bulbar conjunctiva. Corneal sensation was reduced on the right side. The cornea showed a central, large, poorly demarcated, white corneal stromal infiltrate (Figure 1A) involving 50% of the cornea and an overlying epithelial defect measuring 6.8 mm x 8.5 mm (Figure 1B). There was a central corneal perforation with iris tamponade and a slow aqueous leak present. The anterior chamber was flat inferiorly. 

Corneal specimens were obtained by scraping the base and edges of the corneal ulcer using a sterile, disposable 23-gauge needle. Scrapes were collected for gram staining and bacterial, fungal, and acanthamoeba cultures and sensitivities. A PCR sample was acquired by rubbing a sterile cotton bud on the base and edges of the corneal ulcer. The sample was then kept at room temperature before being analyzed in an external laboratory.

Cyanoacrylate glue was applied over the area of perforation, and a 16 mm bandage contact lens was placed over the cornea. After 30 minutes, the right eye was re-examined and the anterior chamber had begun to deepen. Given the reduced corneal sensation, initial raised intraocular pressure, and lack of pain, a clinical diagnosis of HSV keratouveitis was made. She was treated with hourly moxifloxacin 0.5% drops, oral vitamin C tablets, and oral acyclovir 400 mg 5x/day.

Corneal PCR confirmed HSV-1 DNA. Her symptoms improved with treatment, but after three months, the perforation started to leak despite repeat cyanoacrylate gluing. Emergency penetrating keratoplasty was therefore performed, which was successful. Over the last 24 months, she has been taking oral acyclovir 400 mg 2x/day. During this time, the graft has remained clear with no recurrences, and her vision has returned to a 20/30 pinhole.

## Discussion

Diagnosing HSV uveitis can be challenging. This case was diagnosed incorrectly on initial presentation as idiopathic acute anterior uveitis. However, on retrospective analysis, there was sufficient evidence to suggest HSV at the time of presentation. These include raised intraocular pressure, the absence of pain, and the presence of granulomatous keratin precipitates.

HSV ocular disease is commonly caused by HSV-1 [6], and HSV anterior uveitis is more commonly seen during reactivation than in primary diseases [7]. Following primary HSV infection, the HSV-1 genome remains latent within the trigeminal ganglion and can become reactivated following a stress trigger, such as fever [8]. Once reactivation occurs, the HSV-1 genome replicates and is transported down the axon and manifests in a wide variety of ocular presentations, such as blepharitis, conjunctivitis, scleritis, keratitis, and anterior uveitis [4]. 

HSV anterior uveitis tends to be a unilateral disease [9] with a non-specific history of acute redness of the eye associated with photophobia, tearing, and blurred vision. On clinical examination, a prominent ciliary flush is characteristic with cells and flare present in the anterior chamber and granulomatous keratic precipitates [10]. Specific clues to help differentiate HSV anterior uveitis from other non-infectious causes of anterior uveitis include sectoral iris atrophy [11] and raised intraocular pressure [9]. The sectoral iris atrophy was not seen in our case and results from local iris pigment epithelium destruction due to ischaemic necrosis following a previous episode [7]. Raised intraocular pressure and is thought to occur secondary to an active trabeculitis [8]. 

Early misdiagnosis of HSV anterior uveitis is common, and two common differentials considered are Posner-Schlossman syndrome and Fuchs uveitis syndrome. Posner-Schlossman syndrome is characterized by repeated episodes of mild unilateral acute anterior uveitis and very high intraocular pressure [11]. Fuchs uveitis syndrome is associated with less aggressive anterior uveitis, iris heterochromia, vitreous involvement, and cyclitis [12].

While HSV anterior uveitis is a clinical diagnosis, PCR analyses from the cornea and/or aqueous fluid can be performed to confirm the diagnosis. The sensitivity of PCR-based analysis for HSV DNA varies from 40% to 100% in studies [5,13]. Viral cultures are occasionally performed, but due to them being time-consuming and having a low sensitivity [14], they are not that useful in clinical practice. 

In the acute phase of HSV anterior uveitis, oral antiviral agents (acyclovir 400 mg 5xday) and tapering topical corticosteroids are the preferred management. Corticosteroids work to control the anterior chamber inflammation and also help reduce intraocular pressure by targeting the underlying trabeculitis [14]. Very close monitoring is required when tapering the steroid drops to avoid “rebound inflammation,” and this can take many months. Oral acyclovir has been shown to reach therapeutic levels in the ocular surface tear film and aqueous humor [15], thus reducing the need for topical antivirals, even in the presence of herpetic keratitis. Furthermore, topical antivirals alone do not have sufficient penetration to reach the aqueous humor and are also toxic to both the corneal and conjunctival epithelium.

Following an episode of HSV anterior uveitis, a maintenance dose of 400 mg 2xdaily oral acyclovir for at least two years has been proven to reduce the number [6] and duration [16] of recurrence episodes of herpetic anterior uveitis.

## Conclusions

Diagnosing HSV anterior uveitis in the presence of associated dermatitis or dendritic keratitis is simple. However, in the absence of these lesions, it can be more challenging. The three key diagnostic features that are strongly suggestive of anterior uveitis that is secondary to HSV are granulomatous keratic precipitates, raised intraocular pressure, and sectoral iris atrophy. Oral acyclovir and carefully tapering topical corticosteroids are key in the acute phase of management. Long-term oral acyclovir treatment (for a minimum of two years) and close follow-up are essential to decrease the risk of recurrence.
